# Study of Plasma Anti-CD26 Autoantibody Levels in a Cohort of Treatment-Naïve Early Arthritis Patients

**DOI:** 10.1007/s00005-022-00649-6

**Published:** 2022-03-18

**Authors:** Oscar J. Cordero, Irene Viéitez, Irene Altabás, Laura Nuño-Nuño, Alejandro Villalba, Marta Novella-Navarro, Diana Peiteado, María-Eugenia Miranda-Carús, Alejandro Balsa, Rubén Varela-Calviño, Iria Gomez-Tourino, José M. Pego-Reigosa

**Affiliations:** 1 Department of Biochemistry and Molecular Biology, Edificio CIBUS, Universidade de Santiago de Compostela, Campus Vida, 15782 Santiago de Compostela, Galicia Spain; 2 Rare Diseases and Pediatric Medicine Research Group, Galicia Sur Health Research Institute (IIS Galicia Sur), SERGAS-UVIGO, Vigo, Spain; 3 Rheumatology and Immune-Mediated Diseases Research Group (IRIDIS), Galicia Sur Health Research Institute (IISGS), SERGAS-UVIGO, Vigo, Spain; 4 Department of Rheumatology, University Hospital Complex of Vigo-SERGAS, Vigo, Spain; 5 Department of Rheumatology, Hospital Universitario La Paz Research Institute (IDIPAZ), Madrid, Spain; 6 Gene Regulatory Control in Disease Group, Centre for Research in Molecular Medicine and Chronic Diseases (CiMUS), Universidade de Santiago de Compostela, Santiago, Spain; 7 Centre for Research in Molecular Medicine and Chronic Diseases (CiMUS), Universidade de Santiago de Compostela, Santiago, Spain; 8 Health Research Institute of Santiago de Compostela (IDIS), Santiago, Spain

**Keywords:** Autoantibodies, CD26, Anti-CD26, Early rheumatoid arthritis, Autoimmunity, Etiology

## Abstract

In rheumatoid arthritis (RA), the identification of biomarkers to adjust treatment intensity and to correctly diagnose the disease in early stages still constitutes a challenge and, as such, novel biomarkers are needed. We proposed that autoantibodies (aAbs) against CD26 (DPP4) might have both etiological importance and clinical value. Here, we perform a prospective study of the potential diagnostic power of Anti-CD26 aAbs through their quantification in plasmas from 106 treatment-naïve early and undifferentiated AR. Clinical antibodies, Anti-CD26 aAbs, and other disease-related biomarkers were measured in plasmas obtained in the first visit from patients, which were later classified as RA and non-RA according to the American College of Rheumatology criteria. Two different isotype signatures were found among ten groups of patients, one for Anti-CD26 IgA and other for Anti-CD26 IgG and IgM isotypes, both converging in patients with arthritis (RA and Unresolved Undifferentiated Arthritis: UUA), who present elevated levels of all three isotypes. The four UUA patients, unresolved after two years, were ACPA and rheumatic factor (RF) negatives. In the whole cohort, 51.3% of ACPA/RF seronegatives were Anti-CD26 positives, and a similar frequency was observed in the seropositive RA patients. Only weak associations of the three isotypes with ESR, CRP and disease activity parameters were observed. Anti-CD26 aAbs are present in treatment-naïve early arthritis patients, including ACPA and RF seronegative individuals, suggestive of a potential pathogenic and/or biomarker role of Anti-CD26 aAbs in the development of rheumatic diseases.

## Introduction

The American College of Rheumatology (ACR)/European League Against Rheumatism (EULAR) classification criteria was introduced for an earlier identification and treatment of persistent inflammatory arthritis (i.e. rheumatoid arthritis (RA) and undifferentiated arthritis (UA)) (Aletaha et al. 2010; Smolen et al. 2010). The ACR/EULAR classification criteria substituted the original 1987 ACR classification criteria (Nielen et al. 2004), which permitted recruitment of relatively homogeneous patient phenotypes into trials but not the identification of patients with early-stage disease. Evidence now supports better management and outcomes if effective therapy is implemented early (van Dongen et al. 2007), and the range and availability of effective targeted therapies in the clinic is constantly increasing (Kalden 2016).

RA can be subdivided into two major subsets, based on the presence or absence of autoantibodies (aAbs) to citrullinated protein antigens (ACPA) (Aletaha et al. 2010; Smolen et al. 2010). However, to predict which patients will benefit from early intervention with drugs of particular mechanisms of action, what is becoming to be known as pathobiological endotypes (Tarn et al. 2020), and to adjust the treatment intensity, the finding of novel biomarkers still constitutes a challenge (Conti et al. 2020). The course of the disease is similar in seronegative and in seropositive patients at ten years of follow-up (Alivernini et al. 2019).

We recently showed that aAbs against CD26 may have clinical value and may participate in RA pathogenesis (Cordero 2017). In this study, we identified a two-fold increase in Anti-CD26 aAb titers (IgA, IgM and IgG) in serum samples from RA patients under different biological and non-biological therapies, versus healthy donors; ratios with total Ig titers were different for each isotype. These Anti-CD26 aAbs were not ACPA, showed higher titers in smokers and correlated with disease activity parameters such as DAS28. The diagnostic power of these aAbs titers in the group undergoing conventional disease-modifying anti-rheumatic drugs (DMARDs) without biological therapy showed a sensitivity above 80%, and some ACPA negatives were Anti-CD26 positives. In addition, their levels were different when patients were grouped by the type of therapy and they barely correlated with the most commonly used disease activity parameters (ESR, CRP, platelet count, Hb levels or hematocrit).

Recent studies have shown that some aAbs are naturally present in serum, sharing a common ontogeny with conventional antibodies, and that dysregulation of aAb production and function may lead to autoimmune diseases (Sha 2012). The existence of functional aAbs targeting G protein-coupled receptors (GPCRs) in patients with rheumatic diseases has been reported (Cabral-Marques and Riemekasten 2017). Soluble DPP4 (sCD26), is present in the circulation and, in addition to its N-terminal X-Pro cleaving activity (which allows for the regulation of chemotactic responses to inflammatory chemokines), also acts as a neutrophil chemorepellent, by binding to the GPCR protease-activated receptor (PAR)-2 (White et al. 2018). Recently, disease-specific signatures of Anti-GPCR aAb concentrations were observed in sera from patients with several autoimmune diseases, including systemic lupus erythematosus (SLE), granulomatosis with polyangiitis (GPA), RA, systemic sclerosis, and also ovarian cancer and Alzheimer’s disease (Cabral-Marques et al. 2018).

The aim of this proof-of-concept’s study was to analyze the diagnostic power of Anti-CD26 in undifferentiated and treatment-naïve arthritis patients (very early RA: VERA).

## Subjects and Methods

### Study Design

Information about the patients and the procedures for the measurement of serological and clinical scores have already been reported (Orozco et al. 2008; Regueiro et al. 2017). Briefly, patients were recruited between January 1993 and December 2013 (*n* = 104). The entry criteria for the RA clinics were two or more swollen joints for less than a year of onset and the absence of previous treatment with DMARDs. Microcrystalline arthritis, osteoarthritis and infectious arthritis were excluded. Patients were classified at the end of the two-year follow-up period according to the 1987 ACR classification criteria (Regueiro et al. 2017). This classification in RA and non-RA was used as the gold standard.

We defined ten groups of patients, based upon the diagnostic made during the two years of follow-up: (1) RA + polymyalgia rheumatica (PMR) + evolution to RA from palindromic rheumatism (*n* = 38); (2) unresolved undifferentiated arthritis (UUA; *n* = 4); (3) resolved undifferentiated arthritis + palindromic rheumatism (RUA; *n* = 13); (4) related inflammatory processes (RIP): synovitis, Still’s disease, RS3PE, reactive arthritis, and arthralgias (*n* = 7); (5) SLE + one evolved to SLE + Behçet’s syndrome (*n* = 4); (6) chondrocalcinosis/gout (CG; *n* = 5); (7) ankylosing spondylitis (AS; *n* = 8); (8) psoriatic arthritis (PsA; *n* = 5); (9) mechanic pathology, arthritis/tenosinovitis/südeck/arthrosis (MP; *n* = 11); (10) post-infectious arthritis (PIA; *n* = 9). In addition, the plasma of 45 healthy donors were used for comparison.

### Ethic Statement

All the procedures described were performed according to clinical ethical practices of the Spanish and European Administrations and approved by the Local Ethics Committee. The VERA clinic and the sample collection were approved by the La Paz University Hospital Ethics Committee and the Ethics Committee for Clinical Research of Hospital Universitario La Princesa (Ref. PI-518). Written informed consent was obtained from all participants and anonymity was warranted. Healthy donors were recruited from the Agency for the Donation of Organs and Blood (ADOS, Santiago de Compostela, Spain) with the approval of the Director of the Agency and the Clinical Research Ethics Committee of Galicia (2010/298).

### Measurements of Autoantibodies

The levels of clinical antibodies were determined in plasmas obtained in the first visit. The IgM-RF was assessed by nephelometry, whereas anti-CCP antibodies (ACPA) were determined by ELISA. The serological criteria according to the 2010 ACR/EULAR criteria were evaluated (Aletaha et al. 2010; Smolen et al. 2010).

The in-house ELISA for the anti-CD26 isotypes IgG, IgM and IgA has previously been described (De Chiara 2020). Briefly, Anti-CD26 IgA, IgG and IgM titers in plasma were determined by ELISA using 96-well culture plates coated with recombinant sCD26 (rDPP4, 0.5 μg/mL) (RnD Systems, USA) dissolved in phosphate-buffered saline (PBS) pH 7.4 and blocked overnight with PBS 0.5% bovine serum albumin (BSA). Plates were incubated with different dilutions of plasma for 1 h at 37 °C and then washed four times with PBS 0.05% Tween-20. Goat anti-human IgM (μ-chain), anti-IgG (Fab-specific) and anti-IgA (α-specific)-peroxidase conjugates (all from Sigma-Aldrich, USA) were used as detector antibodies, followed by incubation with OPD substrate (o-phenylenediamine dihydrochloride; Sigma-Aldrich, USA) following manufacturer’s instructions. The absorbance at 450 nm was registered using a BioRad Plate reader (BioRad, USA). Data are shown as absorbance units. The specificity of the test has been shown before (Cordero et al. 2017).

### Statistical Analysis

Descriptive statistics were obtained for continuous (mean/median and SD) and categorical variables (frequencies). Differences in anti-CD26 IgG, anti-CD26 IgA and anti-CD26 IgM between two groups were assessed using the parametric Student’s *t* test or the non-parametric Mann–Whitney *U* test. The ANOVA or Kruskal–Wallis test was carried out to compare the variables among more than two groups. Pearson’s correlation was used to evaluate the strength of the linear relationship between the measured variables. Statistical analyses were carried out with the software SPSS version 20 (SPSS, Chicago IL, USA).

## Results

### Anti-CD26 aAbs of Several Isotypes are Found in Plasma of Healthy Donors and Treatment-Naïve Early Arthritis’ Patients

To assess whether Anti-CD26 aAbs were present at baseline in early, treatment-naïve, arthritis patients, we quantified the Ievels of all main isotypes of Anti-CD26 aAbs (IgA, IgG and IgM) in 106 patients at their first visit to the early arthritis clinic. These patients were followed for two years, and in this period a diagnosis was done for all of them. We categorized the patients in ten groups (see Subjects and Methods section and Table 1), based upon their diagnosis after the two-year follow-up period, following the 1987 ACR classification criteria (Regueiro et al. 2017). We also quantified the Anti-CD26 aAbs levels in a cohort of 45 healthy donors.

**Table 1:** Anti-CD26/DPP4 autoantibody levels in plasma of the healthy donor cohort and drug-naïve early arthritis patients

eAR subgroups	Patients number	Ig A		IgG		Ig M	
			95% CI		95% CI		95% CI
RA + PMR + pal	38	0.114 ± 0.121	0.074–0.154	0.277 ± 0.208	0.209–0.345	0.218 ± 0.231	0.141–0.294
UUA	4	0.182 ± 0.276	0.258–0.622	0.316 ± 0.128	0.112–0.520	0.326 ± 0.225	0.033–0.684
RUA	13	0.120 ± 0.146	0.032–0.208	0.227 ± 0.176	0.120–0.333	0.253 ± 0.242	0.107–0.399
RIP	7	0.075 ± 0.064	0.016–0.134	0.192 ± 0.175	0.031–0.354	0.158 ± 0.216	0.042–0.357
SLE	4	0.058 ± 0.043	0.010–0.126	0.240 ± 0.094	0.091–0.389	0.251 ± 0.146	0.020–0.483
CG	5	0.114 ± 0.072	0.025–0.203	0.147 ± 0.064	0.067–0.227	0.106 ± 0.095	0.012–0.224
AS	8	0.068 ± 0.045	0.030–0.106	0.281 ± 0.174	0.136–0.426	0.286 ± 0.307	0.029–0.543
PsA	5	0.068 ± 0.026	0.036–0.099	0.222 ± 0.096	0.104–0.341	0.148 ± 0.171	0.065–0.360
MP	11	0.062 ± 0.050	0.029–0.096	0.154 ± 0.052	0.119–0.189	0.157 ± 0.141	0.063–0.252
PIA	9	0.074 ± 0.048	0.037–0.111	0.368 ± 0.244	0.180–0.556	0.451 ± 0.383	0.157–0.745
HD	45	0.014 ± 0.014	0–0.044	0.117 ± 0.103	0.010–0.333	0.189 ± 0.193	0–0.512

Values in cells represents (mean ± SD of arbitrary absorbance units)

*AS* ankylosing spondylitis, *CG* chondrocalcinosis/gout, *eAR* early arthritis, *HD* healthy donor, *MP* mechanic pathology, *pal* palindromic rheumatism, *PIA* post-infectious arthritis, *PMR* polymyalgia rheumatic, *PsA* psoriatic arthritis, *RA* rheumatoid arthritis, *RIP* related inflammatory processes, *RUA* resolved undifferentiated arthritis, *SLE* systemic lupus erythematosus, *UUA* unresolved undifferentiated arthritis

We found that all the healthy donors present Anti-CD26 aAbs of one isotype or another, with 100% of them having IgG and IgM Anti-CD26 aAbs, and 77.7% also having IgA Anti-CD26 aAbs, albeit at lower titers (Table 1). IgM Anti-CD26 aAb levels are similar to those found in serum in our previous studies, while IgG and, particularly, IgA levels are lower than those in serum (Cordero et al. 2017; De Chiara et al. 2020).

**Fig. 1:**
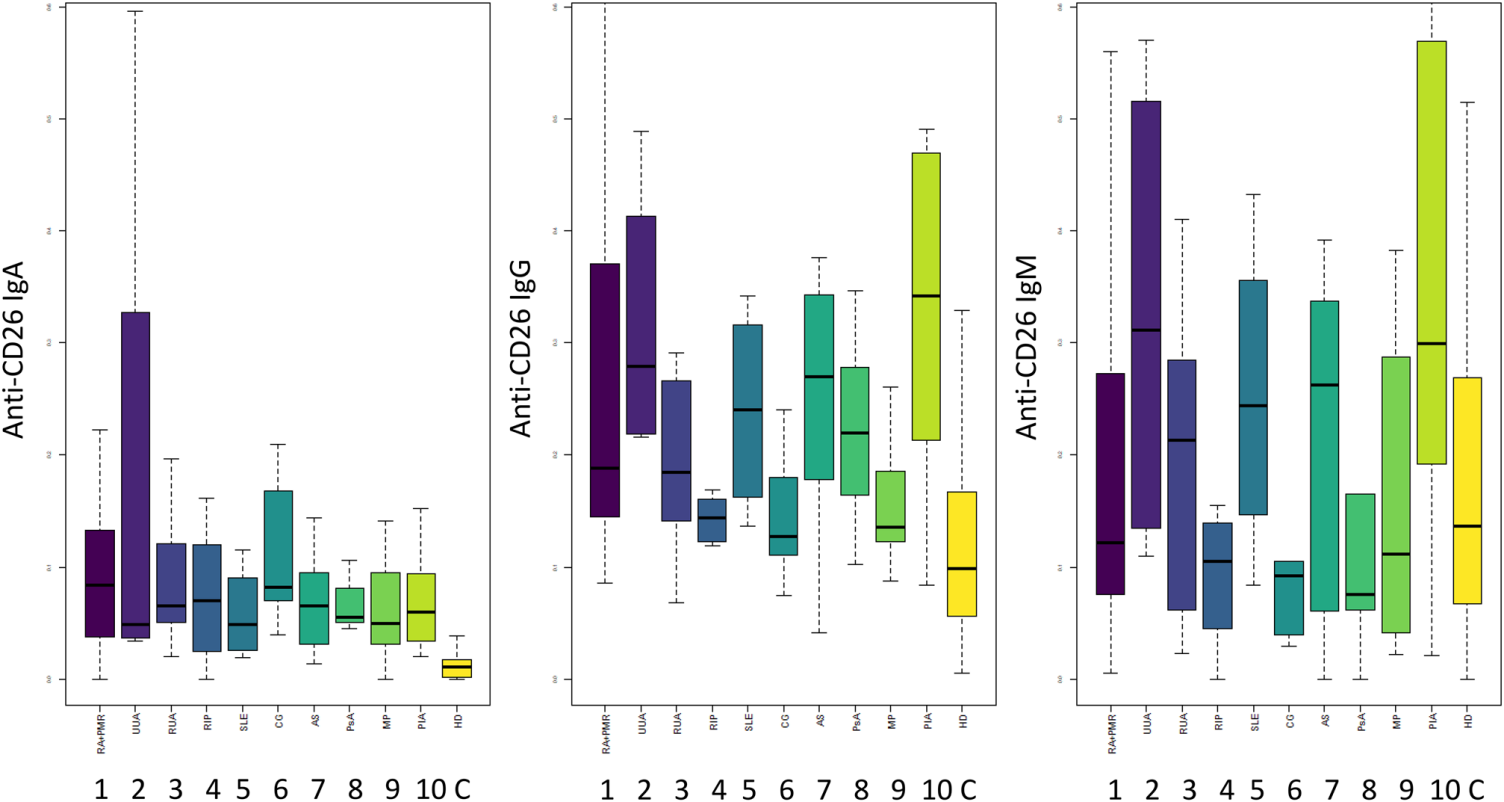
Anti-CD26/DPP4 autoantibody levels in plasma of the healthy donor cohort and drug-naïve early arthritis patients. Values in bars represent median ± SD of arbitrary absorbance units: (1) *RA* rheumatoid arthritis + palindromic rheumatism + PMR: polymyalgia rheumatica; (2) *UUA* unresolved undifferentiated arthritis; (3) *RUA* resolved undifferentiated arthritis; (4) *RIP* related inflammatory processes; (5) *SLE* systemic lupus erythematosus; (6) *CG* chondrocalcinosis/gout; (7) *AS* ankylosing spondylitis; (8) *PsA* psoriatic arthritis; (9) *MP* mechanic pathology; (10) *PIA* post-infectious arthritis; (C) *HD* healthy donor

In the case of the IgA isotype, all groups of patients present higher levels of Anti-CD26 aAbs than healthy donors (Table 1 and Fig. 1), with the highest values found in RA and CG patients; in these two groups of patients, IgA Anti-CD26 aAbs values were around 5- and tenfold higher than those of the other patient groups and healthy donors, respectively. Interestingly, IgG and IgM values in CG patients were the lowest of all the groups, including healthy donors, suggesting a specific Anti-CD26 IgA-specific response in CG.

With respect to the other patient groups, IgG and IgM Anti-CD26 aAb levels were correlated, as expected. The highest median levels were found in UUA and PIA patients, with RA, RUA, SLE, AS and PsA showing intermediate levels, and RIP, CG and MP showing low levels. Interestingly, IgM (but not IgG) levels were lower in these three later groups than in healthy donors.

In summary, we found that Anti-CD26 aAbs are frequent, but with variable levels depending upon the isotype and the disease status.

### Anti-CD26/DPP4 Autoantibody Levels in Plasma with Respect to the ACPA and Rheumatic Factor Positivity

To try to correlate the presence and titer of Anti-CD26 aAbs and disease status, we measured ACPA and rheumatic factor (RF), two markers that are usually detected only in RA patients and are routinely used as diagnostic tools. As expected, we only found one ACPA-positive non-RA patient (RS3PE); interestingly, the other RS3PE patient is RF-positive. Both RS3PE patients lack the Anti-CD26 IgM isotype.

**Fig. 2:**
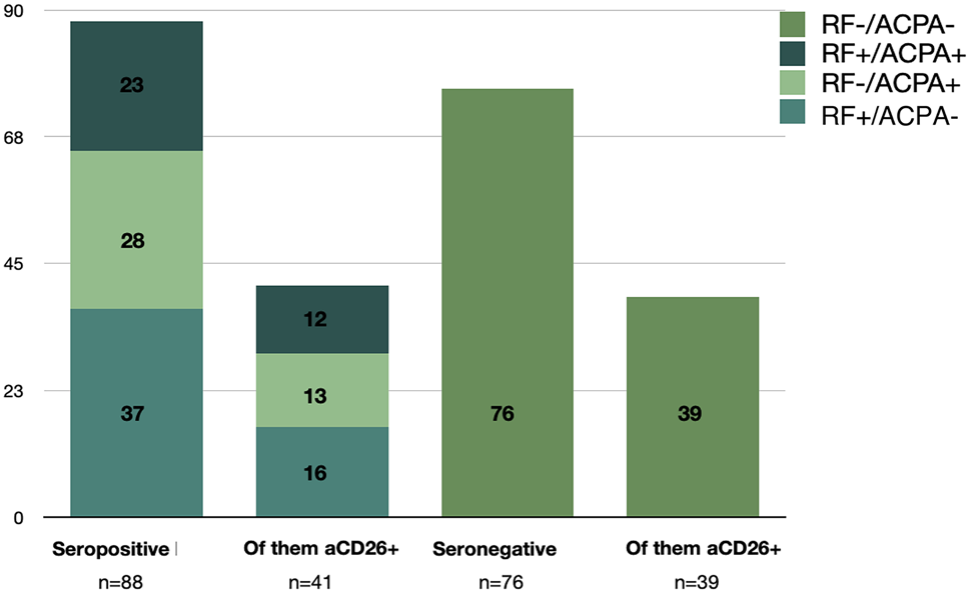
Contingency histogram of the cohort including VERA patients according to the 2010 serological criteria and the Anti-CD26 positivity criteria

From the 38 cases of the RA + PMR + evolution to RA from palindromic rheumatism group, nine RA, two PMR and one palindromic patient were ACPA negative (31.6%), and only three of these nine ACPA-negative RA patients are RF-positive. Strikingly, 50% of the ACPA/RF double negative RA patients showed high levels of Anti-CD26 aAbs. Many seronegative patients (51%) have high titers of Anti-CD26 (Fig. 2). Therefore, we decided to define positivity for Anti-CD26 aAbs employing receiver operating characteristic (ROC) curves.

ROC curves were calculated for each isotype. For IgA isotype, we did not use the healthy donor group values and confronted the four groups with highest levels against the other patients’ titers, all with similar values to the group of MP patients’ titers. For IgG and IgM, we added to the latter group the healthy donor group values and included the AS + PIA + SLE groups in the former. As we focused our clinical interest on sensitivity, the cut-off for positivity was set at the 90% specificity level.

For AUROCs of 0.607, 0.790 and 0.560 respectively, the cut-offs were set in 0.142, 0.205 and 0.372 absorbance arbitrary units for the IgA, IgG and IgM isotypes, respectively. With these cut-offs, three and five healthy donors were positives for the IgG and IgM isotypes, corresponding with a frequency of 6.7 and 11.1%.

For Anti-CD26 IgA isotype, 13/55 of the UUA and RA patients, and 2/5 of the CG patients are positives, with four positives in the other patient groups. One ACPA negative in the RA group is IgA positive. For Anti-CD26 IgG, 26/55 of the UUA and RA patients are positives, with 17/24 positives in the AS + PIA + SLE groups, and two positives in the RIP group. Four ACPA negatives are IgG positives (one is previously mentioned IgA positive). For Anti-CD26 IgM, there are 21 positives overall, many with high titers perhaps reflecting an acute response, 11/55 in the UUA and RA groups, four in PIA and the other six spread among the remainder groups. One ACPA negative is IgM positive, coinciding with one of the IgG positives.

As some of the patient groups are constituted by a low number of individuals, we decided to perform combined group analyses. Therefore, we put together all patients with RA (*n* = 55), RA-related diseases (*n* = 49) and healthy donor (*n* = 45). We managed an algorithm with different cut-offs to achieve the better discrimination. Sorting individuals with IgG cut-off value > 0.14 and from these, sorting individuals with IgA cut-off value > 0.05 as positives, then 33/55 (60%) of the first group, 21/49 (43%) of the second group (*Z* test, *p* = 0.014) and 1/45 of the control group were positives.

The concordant presence of diagnostic RF and APCA autoantibodies improves RA classification among early arthritis patients. Analyzing correlations with Anti-CD26, in the whole cohort, RF and ACPA show strong correlations, highest in the RUA group. However, we only found a very weak trend to correlation between IgA isotype and ACPA (*R* = 0.181). In the groups, whereas in the RA group there is also a similar trend between IgA isotype and the RF (*R* = 0.301, *p* = 0.084), in the RIP group we found a strong negative correlation between IgA isotype and the RF (*R* = − 0.802, *p* = 0.030).

### Correlation of Plasma Anti-CD26 Autoantibody Levels with Disease Activity Parameters

We have previously shown that serum Anti-CD26 aAb levels in treated RA patients with established disease correlated with disease activity parameters, especially with joint damage (Cordero et al. 2017).

In the current study we found, as expected, significant positive correlations between levels of Anti-CD26 IgA and IgG (*R* = 0.633), IgA and IgM (*R* = 0.302), and IgG and IgM (*R* = 0.604) (*p* < 0.001 in all three correlations, Pearson correlation). Similar expected correlations included the positive correlation between DAS28 with swollen (SJC) and tender joint count (TJC) (data not shown). In the whole cohort, no isotype correlates with the disease activity parameters, only a slight trend (*p* < 0.1) for the IgA isotype. With respect to laboratory parameters, IgA isotype positively correlates with the ESR, and IgG and IgM isotypes negatively correlate with the CRP (only IgM) and Hb levels (also IgA) (*p* < 0.05; data not shown).

**Fig. 3:**
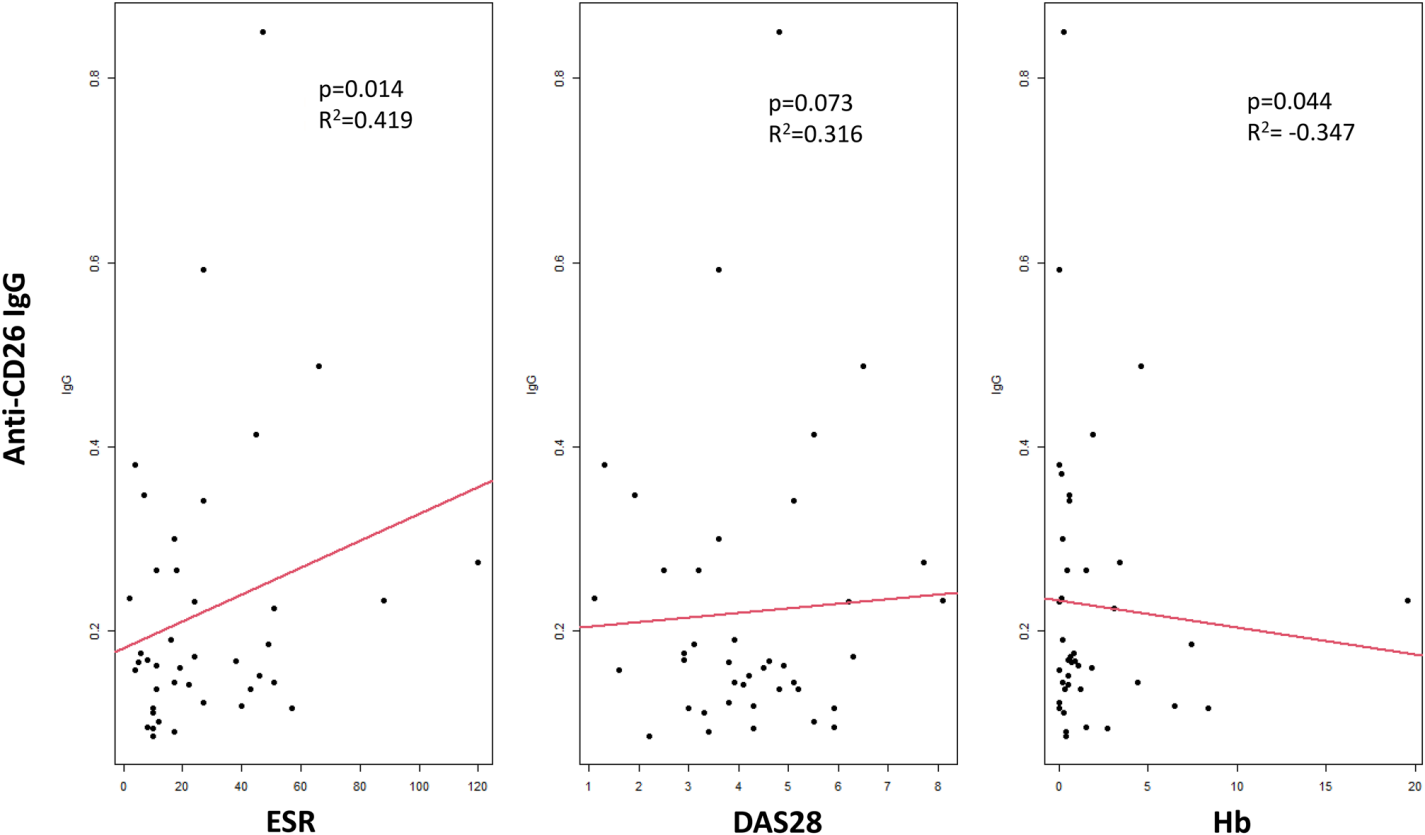
Correlations between disease activity parameters and Anti-CD26 IgG titers in the RA group. Statistically significant correlations between IgG isotype and ESR (**A**) and Hb (**C**) levels but not DAS28 (**B**) were detected in the group of early-RA patients

When correlations are analyzed separately for different patient subgroups, we found a trend for a positive correlation between Anti-CD26 IgG levels and DAS28 in RA patients (Spearman *R* = 0.316, *p* = 0.073) (Fig. 3), and a significant positive correlation with NAD28 in the RIP group (Spearman *R* = 0.782, *p* = 0.038). Interestingly, some of these correlations are of negative sign when non-RA patients are analyzed: Anti-CD26 IgG levels correlate with TJC (Spearman *R* = − 0.419, *p* = 0.003) and SJC (Spearman *R* = − 0.306, *p* = 0.037), while Anti-CD26 IgM levels correlate with DAS28 (Spearman *R* = − 0.320, *p* = 0.032) and TJC (Spearman R = 0.430, *p* = 0.003). The low sample size in some of the non-RA subgroups precludes individual correlation analyses within each subgroup, but the coefficients found are always negative, including the PIA group.

With respect to laboratory parameters, in the RA group Anti-CD26 IgA and IgG isotypes significantly positively correlate with ESR, Anti-CD26 IgG and IgM negatively with Hb, and Anti-CD26 IgM negatively with CRP (Fig. 3). No significant correlations were found between Anti-CD26 aAb levels and laboratory parameters for the non-RA group as a whole. Within the non-RA subgroups, some IgG negative or positive correlations with ESR can be observed, although low patient sample number precludes formal statistical analyses (data not shown).

## Discussion

In RA research, it still remains a challenge to fully understand the complex mechanisms implicated in the loss of immunological tolerance (McInnes and Schett 2017) in the preclinical phase of RA, as the loss of homeostasis between the innate and adaptive arms of the immune system leads to inflammation and pathogenesis of RA, synovial tissue inflammation leading to the onset of joint damage and loss of articular function.

In these events, B-cell derived autoimmunity is relevant, given that aAbs are detected in peripheral blood of asymptomatic subjects up to ten years before the clinical manifestation of the disease. Moreover, B-cell targeted therapy has proved successful in RA treatment (Alivernini et al. 2019; Conti et al. 2020). B cells, in addition to the secretion of aAbs, also modulate both T and dendritic cell function, promote lymphoid neogenesis and release inflammatory mediators (Mauri and Bosma 2012).

In this work, we show altered levels of aAbs against CD26/DPP4 in plasmas of a cohort of treatment-naïve early and undifferentiated arthritis’ patients with respect to healthy donors, as we have previously shown in serum samples from a cohort of treatment-responsive RA patients (Cordero et al. 2017). The levels of the IgA isotype were quite different in plasma and serum; as this did not happen with the other isotypes, this might point to a role of coagulation or complement factors in the measurement. CD26 was found associated to plasminogen (Gonzalez-Gronow et al. 2008), and we have found complexes of sCD26 with the three isotypes of Anti-CD26 (data not shown).

The entry criteria for the early-RA clinics were two or more swollen joints for less than a year and the patients were classified at the end of the two-year follow-up (Regueiro et al. 2017). If we take the levels of mechanic pathology as basal, we found higher levels of Anti-CD26 IgA isotype in the three RA groups and also in the CG group, with the UUA group showing the highest levels. For IgG and IgM, the three RA groups, plus SLE, AS and PIA patients, showed medium-to-high levels of Anti-CD26 aAbs, possibly indicating a relationship among both isotypes.

Most of differences in aAbs levels between groups were not statistically significant due to small sample sizes in some of the patient groups. Therefore, we prepared ROC curves and determined cut-off points to assess for potential clinical value of Anti-CD26 aAbs. We observed that, for Anti-CD26 IgG, 26/55 RA/UUA patients are positives, as well as 17/24 of the AS + PIA + SLE groups. This means that, from a RA point of view, Anti-CD26 aAbs are less RA-specific than ACPA or RF.

However, some ACPA negatives are Anti-CD26 positives. Also, the biological information of Anti-CD26 is not redundant, because whereas the RF and ACPA levels correlated in the cohort, we only found very weak trends to correlation between the IgA isotype and ACPA or the RF. It has been shown that ACPA do not identify all UUA patients who will later progress into developing RA (estimated in about 35–54% of patients included into Large European Early Arthritis cohorts) (Kurowska et al. 2020). In our cohort, the four UUA who remained unresolved after two years from their first visit to the clinic are ACPA- and RF-negative but show very high levels of the three Anti-CD26 isotypes at baseline. Recent studies that included other biomarkers, not only aAbs, have already shown improvements in the identification of RA (Conti et al. 2020; Kurowska et al. 2020; Regueiro et al. 2019), but the sensitivities found for Anti-CD26 aAbs are higher in comparison (Assmann et al. 2020).

We have previously reported, in a cohort of established RA patients under therapies, high sensitivity Anti-CD26 values (Cordero et al. 2017), and serum anti-CD26 levels showed several correlations with disease activity parameters, especially with joint damage (Cordero et al. 2017). In the current study, Anti-CD26 IgG isotype only shows a trend to correlate with DAS28 in the RA group, with no correlation with ACPA. This may be interpreted in two non-incompatible ways: (i) that the Anti-CD26 levels would still raise more with the establishment of the disease; or (ii) that this aAb would contribute to RA pathogenesis. For the first case, in the non-RA groups we found negative correlations for Anti-CD26 IgG and IgM levels with TJC, SJC or DAS28, including the PIA group. Also, the presence of some plasmas with high IgM titers point to a still immature humoral response for some donors. It will be interesting to test the avidity/affinity of the Anti-CD26 IgGs to interpret the maturity, and therefore longevity of the humoral response to this antigen. For the second case, anti-collagen and anti-fibrinogen titers found in the synovial fluid seem related to the articular damage (Amara et al. 2013; Demoruelle et al. 2014). Although Anti-CD26 aAb has not been tested in the synovial fluid, CD26 has been found bound to collagen and fibrinogen and shows collagenase activity in other contexts (Bauvois 1988; Ghersi et al. 2002; Iwase-Okada et al. 1985; Löster et al. 1995; Sánchez-Otero et al. 2014). In addition, myocardial infarction patients who received fibrinolytic therapy developed Anti-CD26 aAbs (Cuchacovich et al. 2002).

In our previous work we described that Anti-CD26 provided different information to the most frequently used disease activity laboratory parameters (ESR, CRP, platelet count, Hb levels or hematocrit) because we only found weak negative correlations of IgG and IgA with the hematocrit and ESR and positive ones with the CRP. In this work, in the RA group the isotypes correlated significantly and positively with the ESR and negatively with the CRP and Hb levels. A similar result was found for the ACPA, a negative trend with CPR and positive correlation with ESR.

In the non-RA patients, as a whole group, a trend to correlation of IgM isotype with CRP levels remained, as well as inside these groups IgG negative or positive correlations or trends with ESR and CRP. These data point to a relationship with reactive acute inflammation, although with a different kinetic according to the type of patient. As mentioned, follow-up for this aAb has not yet been studied.

Anti-CD26 aAbs are naturally present in serum and seem related to other functional aAbs targeting GPCRs (Cabral-Marques and Riemekasten 2017) because its antigen, soluble DPP4 (sCD26) binds to the GPCR PAR-2 at least in neutrophils (White et al. 2018) and monocytes (not shown), and also because they are dysregulated in patients with rheumatic (Cabral-Marques and Riemekasten 2017) and other autoimmune diseases (Shah 2012). The existence of these Anti-GPCRs is being considered a relevant paradigm at the moment. We are working on the many possible regulatory roles of Anti-CD26, as CD26 is a moonlighting protein and its soluble form is present in many biological fluids.

However, the high levels of the Anti-CD26 IgG and IgM isotypes in PIA might point to an additional pathway. PIA is pragmatically considered in the spectrum of infection-related arthritis, in which no viable infective agents can be cultured from the joints. As we previously discussed (Cordero et al. 2017), among the environmental factors triggering autoimmunity in RA, there is a link with periodontal or gingival disease, in particular the oral bacteria *Porphyromonas gingivalis* as the main (but not unique) suspect (Seror et al. 2015). *P gingivalis* expresses deiminase, the enzyme that produces citrulline, perhaps playing a pathogenic role in ACPA-positive patients (Mikuls et al. 2014). As the biofilm formation and production of CD26 are correlated in *P gingivalis*, both being important factors of virulence in periodontitis in the mouse (Clais et al. 2014), and cytokines or bacterial components from *P gingivalis*, *Prevotella intermedia*, and *Escherichia coli* augment the CD26 expression by gingival fibroblasts (Nemoto et al. 1999), we also suggest the possibility of bacterial origin for part of the Anti-CD26 isotypes, at least in some groups. But additional theories are possible, both CD26 and progranulin (Assmann et al. 2020) are adipokines (Fasshauer and Blüher 2015), and Anti-progranulin aAbs have been found in RA patients (Assmann et al. 2020) although not in healthy donors. On the other hand, there is a relationship of Anti-CD26 IgA isotype with the ACPA and RF Abs.

An ongoing study of the epitopes recognized by the Anti-CD26 aAbs may help to differentiate among these origins and their usefulness for protein array-based screening of various rheumatic diseases (Wang et al. 2019). Also, the possible role of the immune complexes of soluble CD26 and its aAbs in the induction of inflammatory response and synovial tissue damage (Ohyama et al. 2011; Szklarski et al. 2021) should be investigated.

## Abbreviations

ACR: American college of rheumatology
EULAR: European league against rheumatism
aAbs: Autoantibodies
RA: Rheumatoid arthritis
VERA: Very early RA
DPP4: Dipeptidyl peptidase 4
ACPA: Antibodies against citrullinated protein antigens
RF: Rheumatic factor
PMR: Polymyalgia rheumatica
UUA: Unresolved undifferentiated arthritis
RUA: Resolved undifferentiated arthritis
RIP: Related inflammatory processes
SLE: Systemic lupus erythematosus
CG: Chondrocalcinosis/gout
AS: Ankylosing spondylitis
PsA: Psoriatic arthritis
MP: Mechanic pathology
PIA: Post-infectious arthritis
DMARDs: Disease-modifying anti-rheumatic drugs
GPCR: G protein-coupled receptors
DAS28: Disease activity score
ESR: Erythrocyte sedimentation rate
CRP: C-reactive protein
ROC: Receiver operating characteristic
TJC: Tender joint count
SJC: Swollen joint count
ELISA: Enzyme-linked immunosorbent assay
PBS: Phosphate-buffered saline
BSA: Bovine serum albumin

## References

[CR1] Aletaha D, Neogi T, Silman AJ (2010). 2010 Rheumatoid arthritis classification criteria: an American College of rheumatology/European league against rheumatism collaborative initiative. Ann Rheum Dis.

[CR2] Alivernini S, Tolusso B, Fedele AL (2019). The B side of rheumatoid arthritis pathogenesis. Pharm Res.

[CR3] Amara K, Steen J, Murray F (2013). Monoclonal IgG antibodies generated from joint-derived B cells of RA patients have a strong bias toward citrullinated autoantigen recognition. J Exp Med.

[CR4] Assmann G, Zinke S, Gerling M (2020). Progranulin-autoantibodies in sera of rheumatoid arthritis patients negative for rheumatoid factor and anti-citrullinated peptide antibodies. Clin Exp Rheumatol.

[CR5] Bauvois B (1988). A collagen-binding glycoprotein on the surface of mouse fibroblasts is identified as dipeptidyl peptidase IV. Biochem J.

[CR6] Cabral-Marques O, Riemekasten G (2017). Functional autoantibodies targeting G protein-coupled receptors in rheumatic diseases. Nat Rev Rheumatol.

[CR7] Cabral-Marques O, Marques A, Giil LM (2018). GPCR-specific autoantibody signatures are associated with physiological and pathological immune homeostasis. Nat Commun.

[CR8] Clais S, Boulet G, Kerstens M (2014). Importance of biofilm formation and dipeptidyl peptidase IV for the pathogenicity of clinical porphyromonas gingivalis isolates. Pathog Dis.

[CR9] Conti V, Corbi G, Costantino M (2020). Biomarkers to personalize the treatment of rheumatoid arthritis: focus on autoantibodies and pharmacogenetics. Biomolecules.

[CR10] Cordero OJ, Varela-Calviño R, López-González T (2017). Anti-CD26 autoantibodies are involved in rheumatoid arthritis and show potential clinical interest. Clin Biochem.

[CR11] Cuchacovich M, Gatica H, Vial P (2002). Streptokinase promotes development of dipeptidyl peptidase IV (CD26) autoantibodies after fibrinolytic therapy in myocardial infarction patients. Clin Diagn Lab Immunol.

[CR12] De Chiara L, Páez de la Cadena M, Rodríguez-Berrocal J (2020). CD26-related serum biomarkers: sCD26 protein, DPP4 activity, and anti-CD26 isotype levels in a colorectal cancer-screening context. Dis Markers.

[CR13] Demoruelle MK, Deane KD, Holers VM (2014). When and where does inflammation begin in rheumatoid arthritis?. Curr Opin Rheumatol.

[CR14] Fasshauer M, Blüher M (2015). Adipokines in health and disease. Trends Pharm Sci.

[CR15] Ghersi G, Dong H, Goldstein LA (2002). Regulation of fibroblast migration on collagenous matrix by a cell surface peptidase complex. J Biol Chem.

[CR16] Gonzalez-Gronow M, Kaczowka S, Gawdi G (2008). Dipeptidyl peptidase IV (DPP IV/CD26) is a cell-surface plasminogen receptor. Front Biosci.

[CR17] Iwase-Okada K, Kojima K, Kato T (1985). Collagenase-like peptidase activity in serum from patients with rheumatoid arthritis. Experientia.

[CR18] Kalden JR (2016). Emerging therapies for rheumatoid arthritis. Rheumatol Ther.

[CR19] Kurowska W, Przygodzka M, Jakubaszek M (2020). Interleukin-15 as a biomarker candidate of rheumatoid arthritis development. J Clin Med.

[CR20] Löster K, Zeilinger K, Schuppan D (1995). The cysteine-rich region of dipeptidyl peptidase IV (CD26) is the collagen-binding site. Biochem Biophys Res Commun.

[CR21] Mauri C, Bosma A (2012). Immune regulatory function of B cells. Annu Rev Immunol.

[CR22] McInnes IB, Schett G (2017). Pathogenetic insights from the treatment of rheumatoid arthritis. Lancet.

[CR23] Mikuls TR, Payne JB, Yu F (2014). Periodontitis and porphyromonas gingivalis in patients with rheumatoid arthritis. Arthritis Rheum.

[CR24] Nemoto E, Sugawara S, Takada H (1999). Increase of CD26/dipeptidyl peptidase IV expression on human gingival fibroblasts upon stimulation with cytokines and bacterial components. Infect Immun.

[CR25] Nielen MM, van Schaardenburg D, Reesink HW (2004). Specific autoantibodies precede the symptoms of rheumatoid arthritis: a study of serial measurements in blood donors. Arthritis Rheumatol.

[CR26] Ohyama K, Ueki Y, Kawakami A (2011). Immune complexome analysis of serum and its application in screening for immune complex antigens in rheumatoid arthritis. Clin Chem.

[CR27] Orozco G, Pascual-Salcedo D, Lopez-Nevot MA (2008). Auto-antibodies, HLA and PTPN22: susceptibility markers for rheumatoid arthritis. Rheumatology.

[CR28] Regueiro C, Nuno L, Ortiz AM (2017). Value of measuring anti-carbamylated protein antibodies for classification on early arthritis patients. Sci Rep.

[CR29] Regueiro C, Rodríguez-Martínez L, Nuño L (2019). Improved RA classification among early arthritis patients with the concordant presence of three RA autoantibodies: analysis in two early arthritis clinics. Arthritis Res Ther.

[CR30] Sánchez-Otero N, Rodríguez-Berrocal FJ, de la Cadena MP (2014). Evaluation of pleural effusion sCD26 and DPP-IV as diagnostic biomarkers in lung disease. Sci Rep.

[CR31] Seror R, Le Gall-David S, Bonnaure-Mallet M (2015). Association of anti-porphyromonas gingivalis antibody titers with nonsmoking status in early rheumatoid arthritis: results from the prospective French cohort of patients with early rheumatoid arthritis. Arthritis Rheumatol.

[CR32] Shah A (2012). The pathologic and clinical intersection of atopic and autoimmune disease. Curr Allergy Asthma Rep.

[CR33] Smolen JS, Landewé R, Breedveld FC (2010). EULAR recommendations for the management of rheumatoid arthritis with synthetic and biological disease-modifying antirheumatic drugs. Ann Rheum Dis.

[CR34] Szklarski M, Freitag H, Lorenz S (2021). Delineating the association between soluble CD26 and autoantibodies against G-protein coupled receptors, immunological and cardiovascular parameters identifies distinct patterns in post-infectious vs non-infection-triggered myalgic encephalomyelitis/chronic fatigue syndrome. Front Immunol.

[CR35] Tarn JR, Lendrem DW, Isaacs JD (2020). In search of pathobiological endotypes: a systems approach to early rheumatoid arthritis. Expert Rev Clin Immunol.

[CR36] van Dongen H, van Aken J, Lard LR (2007). Efficacy of methotrexate treatment in patients with probable rheumatoid arthritis: a double-blind, randomized, placebo-controlled trial. Arthritis Rheum.

[CR37] Wang Y, Liu H, Fu Y (2019). Novel biomarkers containing citrullinated peptides for diagnosis of systemic lupus erythematosus using protein microarrays. Clin Exp Rheumatol.

[CR38] White MJ, Chinea LE, Pilling D (2018). Protease activated-receptor 2 is necessary for neutrophil chemorepulsion induced by trypsin, tryptase, or dipeptidyl peptidase IV. J Leukoc Biol.

